# Angiotensin II: a new approach for refractory shock management?

**DOI:** 10.1186/s13054-014-0694-7

**Published:** 2014-12-18

**Authors:** Antoine Kimmoun, Bruno Levy

**Affiliations:** CHU Nancy, Service de Réanimation Médicale Brabois, Pole Cardiovasculaire et Réanimation Médicale, Hôpital Brabois, 54511 Vandoeuvre les, Nancy, France; INSERM U1116, Equipe 2, Faculté de Médecine, 54511 Vandoeuvre les, Nancy, France; Université de Lorraine, 54000 Nancy, France

## Abstract

Patients with distributive shock still have a high mortality rate and remain an important issue for intensivists. Management of catecholamine-resistant shock in these patients poses a challenging problem. Despite significant advances in the knowledge of its pathophysiology, all innovative therapeutic approaches and interventions have failed to improve outcome. In the previous issue of *Critical Care*, Chawla and colleagues explored the impact of angiotensin II administration in patients with persistent hypotension despite adapted hemodynamic resuscitation. The authors demonstrate that, in case of distributive shock, angiotensin II is an effective vasopressor therapy. Its impact on outcome and adverse effects still needs to be further explored.

In a previous issue of *Critical Care*, Chawla and colleagues tested, in a phase II randomized controlled trial, the hemodynamic effects of angiotensin II, a natural vasopressor hormone without any inotrope or chronotrope effects [1]. In critically ill patients, distributive shock is mainly represented by septic shock [2]. Its mortality rate, after having decreased as the result of the implementation of specific guidelines, still remains unacceptable at about 30% in most recent cohort studies [3]. Vasopressor-refractory hypotension is the last stage before death. Vascular hyporesponsiveness to catecholamines has numerous origins, including nitric oxide overproduction, upregulation of prostacyclin, excessive activation of ATP-sensitive potassium channels and desensitization of alpha adrenoreceptors [4]. Administration of very high doses of catecholamines is a strategy to consider, albeit with many potential side effects such as tachyarrhythmia or myocardial cell damage [5].

The present study of Chawla and colleagues was designed to determine the appropriate dose of angiotensin II in the setting of distributive shock with high cardiac flow. The authors found an almost significant reduction in norepinephrine dosing at a dose range of 1 to 40 ng/kg/minute whereas a rebound of norepinephrine dosing was systematically noticed upon angiotensin II cessation. No clinically relevant adverse effect other than metabolic alkalosis was experienced with angiotensin infusion.

This first clinical trial on the use of angiotensin II in distributive shock raises many questions. Undoubtedly, angiotensin II appears to be an effective vasopressor even if statistical significance was never reached in this trial. Nevertheless, one question should be debated: is there actually a need for a new vasopressor therapy? This intentionally provocative issue reflects the limited literature on the consequences of high catecholamine dosing in refractory shock. First, the maximum tolerable dose is still unknown [6]. Second, even though excessive dosing is associated with tissue hypoperfusion, it is more likely that, in a refractory shock state, adverse ischemic effects are rather related to the shock itself. Finally, in a recent retrospective study, high-dose norepinephrine, particularly in the early phase of septic shock, was not predictive of death [7]. In the present study, patients did not receive high catecholamine dosing. Consequently, patients for whom this new vasopressor therapy could be beneficial were not identified.

Otherwise, no conclusion can be made regarding the adverse effects with only 10 patients included in the angiotensin II group. For the record, recent experimental studies have demonstrated a downregulation of angiotensin 1 receptor-associated protein 1 in animals with septic shock [8]. This downregulation could be an adaptive mechanism to counteract the known pro-inflammatory effects of angiotensin II [9]. In the past, numerous promising vasopressor treatments were ultimately associated with unacceptable adverse effects. For example, the effects of a nitric oxide synthase inhibitor in patients with septic shock were assessed in a phase II, double bind, randomized study [10]. In contrast to the trend toward better outcome in the phase II study, phase III was associated with an increase in overall mortality [11]. This increase in mortality mainly reflected the adverse effects of the treatment on cardiac function. More recently, high hopes have been invested in vasopressin, a vasopressor without any cardiac effects, in order to decrease catecholamine requirements and improve outcome in septic shock patients. In a multicenter, randomized, double-bind trial, adjunction of low-dose vasopressin compared with norepinephrine alone was not associated with any improvement in the 28- or 90-day mortality rate [12]. Interestingly, considering the adverse reactions to vasopressin, patients with a history or coexistent acute coronary syndromes or severe heart failure were excluded from this study. Nonetheless, more patients in the vasopressin group had digital ischemia [12]. Maybauer and colleagues [13] demonstrated that selepressine, a selective vasopressin type 1a receptor agonist, blocks vascular leak more effectively than the mixed vasopressin type 1a receptor/vasopressin V2 receptor agonist arginine vasopressin because of its lack of agonist activity at the vasopressin V2 receptor.

In this work, and as judiciously stated by the authors, the trial was only designed as a proof-of-concept and dose-finding study. For now, it appears unreasonable to judge the safety of this new vasopressor. Further studies, with adapted sample size, are needed to determine the adverse effects of such treatment. Consequently, even though these finding are encouraging, we must be cautious in the definition of the next clinical trials regarding this drug so as not to repeat past failures.
